# Safety and efficacy of prophylactic treatment for hyperthyroidism induced by iodinated contrast media in a high-risk population

**DOI:** 10.3389/fendo.2023.1154251

**Published:** 2023-05-15

**Authors:** Jacopo Manso, Ilaria Piva, Simona Censi, Cristina Clausi, Maria Bardi, Benedetta Schiavon, Isabella Merante Boschin, Francesco Tona, Caterina Mian

**Affiliations:** ^1^ Endocrinology Unit, Department of Medicine (DIMED), University of Padua, Padua, Italy; ^2^ Pediatric Endocrinology Unit, Department of Women’s and Children’s Health, Padua University Hospital, Padova, Italy; ^3^ Cardiology Unit, Department of Cardiac, Thoracic and Vascular Sciences, Padua University Hospital, Padua, Italy; ^4^ Department of Surgical, Oncological, and Gastroenterological Sciences, University of Padova, Padova, Italy

**Keywords:** iodinated contrast media, hyperthyroidism, prophylactic treatment, thyroid, cost-effectiveness

## Abstract

**Introduction:**

The use of iodinated contrast media (ICM) can lead to thyrotoxicosis, especially in patients with risk factors, such as Graves’ disease, multinodular goiter, older age, and iodine deficiency. Although hyperthyroidism may have clinically relevant effects, whether high-risk patients should receive prophylactic treatment before they are administered ICM is still debated.

**Aim of the study:**

We aimed to demonstrate the safety and efficacy of prophylactic treatment with sodium perchlorate and/or methimazole to prevent ICM-induced hyperthyroidism (ICMIH) in a population of high-risk cardiac patients. We ran a cost analysis to ascertain the most cost-effective prophylactic treatment protocol. We also aimed to identify possible risk factors for the onset of ICMIH.

**Materials and methods:**

We performed a longitudinal retrospective study on 61 patients admitted to a tertiary-level cardiology unit for diagnostic and/or therapeutic ICM-procedures. We included patients with available records of thyroid function tests performed before and after ICM were administered, who were at high risk of developing ICMIH. Patients were given one of two different prophylactic treatments (methimazole alone or both methimazole and sodium perchlorate) or no prophylactic treatment. The difference between their thyroid function at the baseline and 11-30 days after the ICM-related procedure was considered the principal endpoint.

**Results:**

Twenty-three (38%) of the 61 patients were given a prophylactic treatment. Thyroid function deteriorated after the administration of ICM in 9/61 patients (15%). These cases were associated with higher plasma creatinine levels at admission, higher baseline TSH levels, lower baseline FT4 levels, and no use of prophylactic treatment. The type of prophylaxis provided did not influence any onset of ICMIH. A cost-benefit analysis showed that prophylactic treatment with methimazole alone was less costly per person than the combination protocol. On multivariate analysis, only the use of a prophylactic treatment was independently associated with a reduction in the risk of ICMIH. Patients not given any prophylactic treatment had a nearly five-fold higher relative risk of developing ICMIH.

**Conclusion:**

Prophylactic treatment can prevent the onset of ICMIH in high-risk populations administered ICM. Prophylaxis is safe and effective in this setting, especially in cardiopathic patients. Prophylaxis with methimazole alone seems to be the most cost-effective option.

## Introduction

The use of imaging techniques that involve administering iodinated contrast media (ICM) is expanding considerably (1, 2). The most frequent adverse effects of ICM are nephropathy, extravasation of contrast media, thrombosis, and acute reactions (3–5).

ICM generally contain about 320 to 370 mg/mL of iodine, an amount far above the recommended daily intake (6, 7). Their administration does not usually cause thyroid dysfunction in the general population. It can induce thyrotoxicosis, however, especially in people with known risk factors, such as Graves’ disease, multinodular goiter, older age and iodine deficiency. Although ICM are known to be a potential cause of hyperthyroidism, there is a paucity of evidence regarding the real prevalence of this adverse effect and the value of prophylactic treatment to prevent it (8–11).

The prevalence of ICM-induced hyperthyroidism (ICMIH) was reportedly between 0.05% and 5% in various studies on unselected populations (12). A recent meta-analysis estimated the prevalence of overt hyperthyroidism after the administration of ICM and found an extremely low figure (0.1%) in the general population, but most of the studies considered did not examine potential risk factors for the onset of hyperthyroidism. When studies conducted in regions where goiter is endemic, or on older populations, or samples with a greater frequency of thyroid nodules were considered separately, the observed prevalence of hyperthyroidism was far from negligible (between 10-15%) (13, 14).

Soon after an iodine load, thyroid organification processes and hormone synthesis stop as a defense mechanism to avoid hyperthyroidism and thyroid toxicosis (the so-called “Wolff-Chaikoff effect”). After 24-48 hours, the healthy thyroid escapes this Wolff-Chaikoff effect and thyroid hormone synthesis returns to normal (5, 15). An existing thyroid disease can impair normal thyroid response to an iodine load, leading to iodine-induced hypo- or hyperthyroidism. Subclinical or overt hyperthyroidism develops more frequently in patients with latent or known autonomous thyroid tissue (thyroid nodules or Graves’ disease, for example), leading to the so-called “Jod-Basedow phenomenon” (16–19). Hyperthyroidism may raise mortality and morbidity rates due to its cardiovascular effects, particularly in frail cardiological patients (20–23).

The latest European Thyroid Association (ETA) guidelines do not recommend thyroid function screening before radiological exams in the general population due to the low prevalence of ICMIH. They do suggest thyroid function tests before administering ICM to subjects at risk of thyroid dysfunction, however, especially in the elderly and people at risk for concomitant cardiovascular disease (24). Hyperthyroidism can have clinically relevant consequences, but the issue of whether or not patients at high risk of hyperthyroidism who require procedures involving ICM should be given prophylactic treatment is still debated (24). The ETA guidelines suggest prophylactic treatment only for selected patients more prone to the negative effects of ICMIH, such as the elderly with persistent endogenous subclinical hyperthyroidism and/or nodular goiter, and/or concomitant cardiovascular disease, especially in areas with iodine deficiencies. That said, the guidelines do not recommend any specific prophylactic drug. Different protocols have been proposed, involving various combinations of thyrostatic drugs and sodium perchlorate. Though some studies suggest that combined treatments are more effective, there is still no strong evidence to support the safety and efficacy of the different protocols (4, 8, 24–28).

The present study aimed to demonstrate the safety and efficacy of prophylactic treatment with sodium perchlorate and/or methimazole, in preventing ICMIH in a population of high-risk patients suffering from various cardiological diseases. We also ran a cost-benefit analysis to ascertain the most cost-effective prophylactic treatment protocol, and tried to identify possible risk factors for the onset of ICMIH.

## Materials and methods

### Patients

This longitudinal retrospective study included patients admitted to a tertiary-level Cardiology Unit at Padua University Hospital between 2020 and 2022 who underwent diagnostic and/or therapeutic procedures involving ICM and needed an endocrinological assessment.

Inclusion criteria were: i) patients over 65 years old admitted to the Cardiology Unit for procedures relating to cardiovascular diseases that involved the intravenous administration of ICM (e.g. coronary angiography, angiography, transcatheter aortic valve implantation, computed tomography with contrast media), who were at risk of developing ICMIH due to multinodular goiter, Plummer’s adenoma, Graves’ disease, subclinical hyperthyroidism (defined as thyroid-stimulating hormone [TSH] <0.5 mIU/L with normal thyroid hormone levels according to local reference values), overt hyperthyroidism (defined as TSH <0.1 mIU/L and high circulating thyroid hormone levels according to local reference values); ii) patients with available results of thyroid function tests conducted before and 11-30 days after the administration of ICM. Exclusion criteria were: i) patients administered ICM within 90 days before admission to the cardiology unit; ii) ongoing or former use of drugs interfering with thyroid function (amiodarone, glucocorticoids, levothyroxine, positive inotropic agents). Based on our selection criteria, we enrolled 61 patients, and 54 of them had already known thyroid disease according to their medical history. Patients’ clinical, biochemical and imaging data were collected from their medical records.

The contrast media administered were: iodixanol (Visipaque 320), iohexol (Omnipaque350) and iobiditrol (Xenetix350).

### Laboratory tests

Electrochemiluminescence immunoassay (ECLIA) platforms were used to measure serum concentrations of TSH, free thyroxine (FT4), free triiodothyronine (FT3) (Roche, Mannheim, Germany), and TSH-receptor autoantibodies (Trab) (Maglumi^®^, Snibe Diagnostics, China). Plasma creatinine levels were measured on admission using ECLIA (Roche, Cobas C702).

The normal ranges, analytical sensitivities and intra- and inter-assay coefficients of variation were:

TSH: 0.27–4.2 mIU/l; 0.005 mIU/l; 3% and 8%;
FT3: 3-6 pmol/l; 0.6 pmol/l; 3.5% and 3.6%;
FT4: 12–22 pmol/l; 0.30 pmol/l; 2% and 5%;
TRAB: <1.5 IU/L; <0.28 IU/L; 4% and 3%;
creatinine: 45-84 µmol/L (female), 59-104 µmol/L (male);
< 5 µmol/L; 1.1% and 1.4%.

Thyroid function tests were performed before and after the first administration of ICM. Laboratory data were collected and subdivided at preset times after the first ICM-related procedure: after 4-10 days, 11-30 days, and 30-90 days.

### Clinical assessments

Weight, height and body mass index (BMI) were obtained from the patients’ medical records.

### Prophylactic treatment protocols

Patients newly given sodium perchlorate and/or methimazole, and those already taking methimazole before admission who were given higher doses of methimazole before ICM were administered, were classified as “treated” for prophylactic purposes. Patients not given any thyrostatic agents, and those already on methimazole therapy who maintained the same dose, without any addition of sodium perchlorate, before any ICM were administered were classified as “untreated” for prophylactic purposes. Our treated patients were non-randomly assigned to receive one of two different prophylactic treatments, as prescribed by clinicians on the basis of their clinical history, biochemical data and imaging studies. The two options were: 1) methimazole alone; or 2) methimazole plus sodium perchlorate. The dosage of methimazole varied, depending on the severity of patients’ hyperthyroidism and/or any existing methimazole therapy: i) if the patient was already in methimazole therapy and in euthyroidism we increased the dose by 100% starting from 24 h before the ICM administration; ii) if the patient was already in methimazole therapy but in subclinical hyperthyroidism we increased the dose between 150-250% starting from 24 h before the ICM administration; iii) if the patient was already in methimazole therapy but in overt hyperthyroidism we increased the dose between 200-300% starting from 24 h before the ICM administration; iiii) if the patient was in subclinical or overt hyperthyroidism and naïve for any treatment, the methimazole starting dose depends from a clinical judgment (FT4 and FT3 blood levels and comorbidities). While sodium perchlorate was always 600 mg/daily, divided into three 200 mg doses (10 drops each), starting 24 hours before the ICM were administered, and continuing for a minimum of 7 days to a maximum of 15 days.

The methimazole therapy was progressively tapered with weekly checks based on the laboratory test and clinical status. None of the patients discontinued methimazole treatment during follow-up.

The study was conducted in accordance with the Declaration of Helsinki. All data were collected retrospectively, and all tests were performed as part of standard patient care, so this study did not need ethical committee approval.

### Statistical analysis

The statistical analysis was performed using MedCalc (version 18.11.3) software. The Kolmogorov-Smirnov test was used to assess the normal distribution of all variables. All data were expressed as means ± standard deviations (SD) for variables that were normally distributed, and as medians with interquartile ranges (IQR) for those that were not. We obtained 4 measurements for each patient during follow up, however we chose the difference between baseline thyroid function and 11-30 days thyroid function after ICM as the primary endpoint for thyroid function assessment.

When a dichotomized variable was needed for statistical purposes, patients were grouped as follows, by considering thyroid function 11-30 days after exposure to ICM in comparison with their baseline values:

- “worsened” if their thyroid function had deteriorated (higher fT4 and/or fT3 levels and/or lower TSH levels); or

- “stable/improved” if their thyroid function was stable or better (unchanged or higher TSH levels, unchanged or lower fT4 and fT3 levels). Some statistical analyses demanded clustering of the thyroid function 11-30 days after exposure to ICM by comparison with the baseline value in three groups, as follows:

- “worsened” if patients’ thyroid function had deteriorated (higher fT4 and/or fT3 levels and/or lower TSH levels);

- “stable” if their thyroid function remained stable (unchanged TSH, fT4 and fT3 levels);

- “improved” if thyroid function improved (higher TSH levels and/or lower fT4 and/or fT3 levels). The Mann-Whitney, χ^2^ and Kruskal-Wallis (with Conover’s *post-hoc* analysis) were used, as appropriate, to compare the clinical and biochemical data between patients who did and those who did not receive prophylactic treatment. The same tests were used to compare their clinical and biochemical features to assess the primary endpoint in univariate analysis. We also ran a statistical analysis of the relative risk of ICMIH developing in treated as opposed to untreated patients.

All results were considered statistically significant when p <0.05.

## Results

**Table 1** shows patients’ clinical and biochemical features. The study sample included 61 subjects, 35 females (57%), and 26 males (43%), who were a median 72 years old (range 66-80 years) on admission. Twenty-three (38%) of the 61 patients were given prophylactic treatment, at their clinician’s discretion, with methimazole alone or sodium perchlorate plus methimazole before their ICM-related procedure. The treated and untreated groups did not differ significantly in terms of age, sex, Trab levels, plasma creatinine, or total administered iodine. Patients who were given prophylaxis had a lower BMI (p= 0.03), lower TSH levels (p=0.001), and higher FT4 levels (p=0.003) than those who were not (**Table 2**).

**Table 1:** Clinical and biochemical features of patients involved in the study.

	N (%)
Age (range)	72 years [66-80.00]
Sex	
male	26 (43%)
female	35 (57%)
Height (median; [IQR])	166.00 cm [160.00-175.00]
Weight (mean ± SD)	73.64 kg ± 13.88
BMI (mean ± SD)	26.35 kg/m^2^ ± 4.22
Thyroid disease	
Graves’ disease	17 (28%)
multinodular goiter	37 (61%)
Plummer’s adenoma	4 (6%)
subclinical/overt hyperthyroidism of unknown origin	3 (5%)
Basal TSH (median; [IQR])	0.51 mIU/L [0.05-1.42]
Basal fT4 (median; [IQR])	17.05 pmol/L [13.91-21.58]
Basal fT3 (median; [IQR])	4.22 pmol/L [3.52-5.33]
TSH after 4-10 days (median; [IQR])	0.63 mIU/L [0.02-1.89]
fT4 after 4-10 days (median; [IQR])	17.51 pmol/L [14.22-19.11]
fT3 after 4-10 days (mean ± SD])	4.02 pmol/L ± 1.36
TSH after 11-30 days (median; [IQR])	0.21 mIU/L [0.02-0.94]
fT4 after 11-30 days (mean ± SD)	16.49 pmol/L ± 5.13
fT3 after 11-30 days (mean ± SD)	3.95 pmol/L ± 1.42
TSH after 30 days (median; [IQR])	1.63 mIU/L [0.68-3.38]
fT4 after 30-90 days (median; [IQR])	14.87 pmol/L [11.16-16.67]
fT3 after 30-90 days (median; [IQR])	4.07 pmol/L [3.68-5.14]
Trab (median; [IQR])	4.50 IU/L [2.68-7.30]
Plasma Creatinine (median; [IQR])	74.00 µmol/L [59.00-95.25]
Total iodine administered (median; [IQR])	51200 mg [32175-55319]

**Table 2:** Baseline clinical and biochemical characteristics of patients who were versus were not given prophylactic treatment.

	Prophylaxis group N=23	Untreated group N=38	p
Age (median; [IQR])	71 years [64-81]	72 years [67-80]	0.86
Sex			
male	11 (42%)	15(58%)	0.53
female	12 (34%)	23 (66%)	
**BMI (mean ± SD)**	**24.8 kg/m^2^ ± 3.9**	**27.4 kg/m^2^ ± 4.2**	**0.03**
**Basal TSH (median; [IQR])**	**0.05 mIU/L [0.01-0.74]**	**1.04 mIU/L [0.21-2.12]**	**0.001**
**Basal fT4 (median; [IQR])**	**19.8 pmol/L [16.89-26.68]**	**15.58 pmol/L [13.63-18.61]**	**0.003**
Basal fT3 (median; [IQR])	5.19 pmol/L [3.56-6.85]	4.12 pmol/L [3.58-4.52]	0.09
Trab (median; [IQR])	4.4 IU/L [3.8-5.3]	4.5 IU/L [2.2-13.2]	0.94
Plasma creatinine (median; [IQR])	73.00 µmol/L [56-88]	76.00 µmol/L [62-100]	0.44
Total administered iodine (median; [IQR])	44800 mg [37200-59937]	54250 mg [37760-64400]	0.67

Lines in bold highlight statistical significance.

Regarding the prophylactic treatment, only one patient (4%) was given sodium perchlorate alone (600 mg daily for 13 days), 5 (22%) were given methimazole alone (median daily dose 10 mg, IQR 8.44-22.5), and 17 (74%) received sodium perchlorate (median daily dose 600 mg, IQR 600-600, lasting for a median of 10 days IQR 7.0-14.3) plus methimazole (median daily dose 10 mg, IQR 5-15). There were no drug-related adverse effects. One patient died: he had a known toxic multinodular goiter and overt hyperthyroidism on admission; he was given prophylactic treatment with methimazole and sodium perchlorate starting 2 days before the ICM-related procedure, with an improvement in his thyroid function; his death was due to a worsening of his preexisting cardiovascular disease.

### Univariate analysis

When tested 11-30 days after administering ICM, thyroid function was stable in 57% of patients (35/61), improved in 28% (17/61), and worsened in 15% (9/61). Among all the variables considered, univariate analysis indicated that a deterioration in thyroid function was associated with higher plasma creatinine levels on admission (p=0.03), with higher baseline TSH levels (p< 0.0001) and lower baseline FT4 levels (p<0.01), and with the lack of administration of prophylactic treatment (p=0.01) (**Table 3**).

**Table 3:** Univariate analysis of risk factors for worsening/improving thyroid function after administration of contrast media.

	Stable n=35	Improved n=17	Worsened n=9	p
Age (median; [IQR])	71 years [66-80]	80 years [65-84]	68 years [67-77]	0.17
Sex				0.77
male	16 (62%)	6 (23%)	4 (15%)	
female	19 (54%)	11 (32%)	5 (14%)	
BMI (median; [IQR])	27.80 kg/m^2^ [23.55-31.00]	24.60 kg/m^2^ [22.53-29.15]	25.90 kg/m^2^ [23.35-26.98]	0.22
Total administered iodine (median; [IQR])	51200 mg [41600-58400]	54250 mg [35850-69200]	37280 mg [35200-78400]	0.64
**Plasma creatinine (median; [IQR])**	76.00 µmol/L [68.00-94.25]	59.00 µmol/L [54.75-72.75]	100.00 µmol/L [70.50-112.50]	**0.03**
**Basal TSH (median; [IQR])**	1.04 mIU/L [0.24-1.47]	0.02 mIU/L [0.01-0.08]	1.13 mIU/L [0.45-2.24]	**< 0.0001**
**Basal fT4 (median; [IQR])**	16.45 pmol/L [13.69-19.00]	23.00 pmol/L [17.13-28.22]	14.77 pmol/L [12.09-18.50]	**<0.01**
Basal fT3 (median; [IQR])	4.23 pmol/L [3.27-4.85]	5.69 pmol/L [3.66-7.81]	4.06 pmol/L [3.76-4.26]	0.09
Total days of sodium perchlorate prophylaxis (median; [IQR])	13.00 days [9.00-15.50]	7.00 days [7.00-11.00]	9.00 days [7.50-9.75]	0.09
Total days of methimazole prophylaxis (median; [IQR])	12.00 days [7.00-23.50]	14.00 days [10.75-20.00]	15.00 days [10.50-23.25]	0.91
Sodium perchlorate daily dose (median; [IQR])	600.00 mg [600.00-600.00]	600.00 mg [600.00-600.00]	600.00 mg [600.00-600.00]	0.63
**Prophylactic treatment before administration of contrast media**				**0.01**
yes	13 (57%)	9 (39%)	1 (4%)	
no	22 (58%)	8 (21%)	8 (21%)	

Lines in bold highlight statistical significance.

When we considered only patients (n=35) already being treated with methimazole for thyroid disease before admission, we found that patients who were given prophylactic treatment (n=10, 29%) prior to the administration of ICM seemed to develop ICMIH less frequently than those who were not, although the difference did not reach statistical significance (p=0.06).

In the group of patients treated for prophylactic purposes, the different protocols (sodium perchlorate plus methimazole versus methimazole alone) did not affect final thyroid function (p=0.42). The same was true in naive patients who only started thyrostatic treatment for prophylaxis prior to their procedure involving ICM (p=0.57).

**Figure 1:**
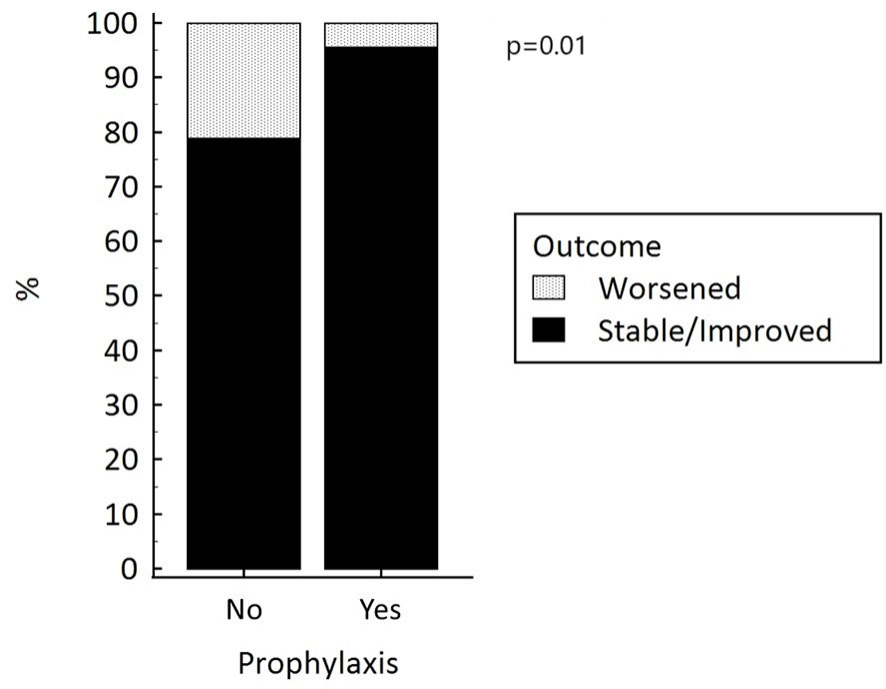
Thyroid function after the administration of iodinated contrast media, with and without prophylactic treatment to prevent hyperthyroidism.

To run a multivariate analysis to identify independent risk factors for the onset of ICMIH, we dichotomized patients’ primary endpoint in terms of a “worsened” versus “stable/improved” thyroid function after the administration of ICM (**Table 4**). Only the lack of any administration of prophylactic treatment independently predicted a worsening thyroid function (p=0.01), **Figure 1**. Patients not given prophylactic treatment were more likely to have a worsened thyroid function at follow-up: they carried a nearly five-fold higher relative risk of developing ICMIH (RR 4.8; IC 95% 1.2-20.1; p=0.03). Among the patients given prophylactic treatment, the type of treatment had no influence on the primary endpoint, not even when it was dichotomized (p=0.69).

**Table 4:** Univariate analysis of risk factors for hyperthyroidism induced by contrast media.

	Worsened n=9	Stable/improved n=52	p
Age (median; [IQR])	68 years [67-77]	73 years [66-81]	0.26
Sex			0.90
male	4 (15%)	22 (85%)	
female	5 (14%)	30 (86%)	
BMI (median; [IQR])	25.90 kg/m^2^ [23.35-26.98]	26.30 kg/m^2^ [23.20-30.00]	0.52
Total administered iodine (median; [IQR])	37280 mg [35200-78400]	52725 [40000- 61025]	0.35
Plasma creatinine (median; [IQR])	100.00 µmol/L [70.50-112.50]	72.00 µmol/L [59.00-90.50]	0.16
Total administered iodine (median; [IQR])	37280 mg [35200-78400]	52725 mg [40000-61025]	0.37
Basal TSH (median; [IQR])	1.13 mIU/L [0.45-2.24]	0.32 mIU/L [0.04-1.27]	0.07
Basal FT4 (median; [IQR])	14.77 pmol/L [12.09-18.51]	17.16 pmol/L [14.47-23.42]	0.11
Basal FT3 (median; [IQR])	4.06 pmol/L [3.76-4.26]	4.31 pmol/L [3.51-5.67]	0.32
Total days of sodium perchlorate prophylaxis (median; [IQR])	9.00 days [7.50-9.75	10.00 days [7.00-14.00]	0.46
Total days of methimazole prophylaxis (median; [IQR])	15.00 days [10.50-23.25]	14.00 days [7.75-20.00]	0.73
Sodium perchlorate daily dose (median; [IQR])	600.00 mg [600.00-600.00]	600.00 mg [600.00-600.00]	0.70
**Prophylactic treatment before contrast media administration**			**0.01**
yes	1 (4%)	22 (96%)	
no	8 (21%)	30 (79%)	

Lines in bold highlight statistical significance.

### Cost-effectiveness analysis on the different prophylactic treatments

Since the two prophylactic treatment options did not differ in terms of efficacy, and no treatment-related adverse events were reported for any of them, we ran a cost analysis to identify the most cost-effective option for preventing ICMIH. As only one patient was given sodium perchlorate alone, only patients treated with methimazole alone, and those given methimazole plus sodium perchlorate were considered in our analysis.

1. The cost of methimazole alone (given to 5 patients) was calculated from a 5 mg pill price of 0.058 Euros (€), and the median total cost of this treatment per person was 3.48 €, calculated as follows:

median days of prophylactic treatment x mean daily dose

20 days (IQR 19-27) x 15 mg (± SD 10 mg) = 20 days x 15 mg = 20 days x 3 cp = 20 days x (0.058 € x 3) = 3.48 €.

2. The cost of methimazole plus sodium perchlorate, administered to 17 patients, was computed as follows because sodium perchlorate is available in 40 mL bottles (300 mg/mL), priced at 12.50 €, and administered in 600 mg daily doses:

(median days of prophylactic treatment with methimazole x mean daily dose of methimazole) + (median days of prophylactic treatment with sodium perchlorate x mean daily dose of sodium perchlorate)

[13 days (IQR 8-20) x 11 mg (± SD 7 mg)] + [10 days (IQR 7-14) x 600 mg (IQR 600-600 mg)] = [13 days x 2.2 cp] + [10 days x 2 mL] = 1.66 € + 6.25 € =7.91 €.

Prophylactic treatment with methimazole alone was less costly per person than the combination therapy.

## Discussion

A non-negligible number of cases of hyperthyroidism occur after the administration of ICM in patients at risk, such as those with Graves’ disease or thyroid nodules, with non-negligible clinical consequences - especially in fragile, old and cardiopathic patients (29). Although ICMIH does not seem to have a relevant clinical impact on the general population, it is advisable to assess thyroid function before and after administering ICM to patients at high-risk of developing hyperthyroidism, or in whom the effects of ICMIH can have severe clinical consequences, and prophylactic treatment may be warranted (8, 24, 28). In a previous study, Bonelli et al. found a 10% risk of hyperthyroidism after ICM in a group of 810 consecutive patients with cardiovascular comorbidities, although 42% of their patients had already been diagnosed with hyperthyroidism beforehand (30). The potential benefit of prophylaxis against ICMIH with drugs like methimazole and/or sodium perchlorate has already been investigated, but few studies were performed on populations of patients with cardiovascular disease administered ICM for coronary angiographies. A German randomized clinical trial conducted on a series of 51 subjects in 1996 found that medical prophylaxis with sodium perchlorate or methimazole had a protective effect in patients with thyroid autonomy (26). A prospective study published in 2004 found that thyroid scintigraphy using Tc-99m-pertechnetate can predict the onset of ICMIH in patients with low basal TSH levels: patients with a low thyroid volume and a low uptake on scintigraphy are at low risk of ICMIH, and probably do not require prophylactic therapy (27, 28).

Although risk factors for ICMIH have been described, no clear recommendations have been provided on the indications for prophylaxis and the type of treatment to administer (24). Few studies, only one of them randomized, have investigated the efficacy of such treatments, and different protocols have been proposed, involving non-homogeneous types of medication (methimazole alone versus methimazole plus perchlorate), dosages, and durations of treatment.

The peculiarity of our study lies in that we considered subjects at high risk of developing ICMIH, as they were over 65 years old, and had multinodular goiter or Graves’ disease, or presented with subclinical or overt hyperthyroidism on admission. We thus aimed to investigate the safety and efficacy of prophylaxis in a real-life clinical setting. In fact, overt hyperthyroidism would contraindicate the use of ICM, but in emergencies, as occurring in a Cardiology Unit, it is pivotal to understand how to prevent worsening of hyperthyroidism when contrast media administration can’t be postponed. Hence our inclusion of patients with cardiac morbidities and thyroid disease, in whom the onset of hyperthyroidism can potentially lead to severe, even life-threatening clinical complications.

Our treatment protocol included both methimazole and sodium perchlorate in lower dosages than those used in previous studies. There were no drug-related adverse effects recorded in our series, whether these drugs were used alone or in combination.

We chose the difference between 11-30 days thyroid function in comparison to baseline values as our principal endpoint. Notably, thyroid function evaluation at 30 day was the most frequently adopted timepoint in most of the previous studies. In fact, the onset of overt hyperthyroidism after exposure to ICM is supposed around that time, while urinary iodine excretion usually normalizes within 30-40 days (24, 31).

We found that prophylaxis had a significant effect in protecting against a worsening thyroid function after the administration of ICM (achieving a five-fold lower relative risk). This would also apply to patients already receiving thyrostatic therapy for known thyroid disease, although our findings did not reach the full statistical significance on this aspect (probably due to the small size of our sample). This is consistent with a previous report on a Polish population of 36 patients with euthyroid goiter who underwent procedures involving ICM, that found a significant reduction in the onset of ICMIH in the group given prophylactic treatment with thiamazol, either in monotherapy or in combination with sodium perchlorate (15% vs 65% for untreated patients) (28).

We almost did not observe any worsening or new-onset hyperthyroidism among our patients treated prophylactically, regardless of the type of treatment administered. It is worth noting that our treated group had lower TSH and higher FT4 levels at the baseline than the untreated group, which is further proof of the efficacy of the prophylactic treatment. Lower TSH levels and higher FT4 levels at the baseline were actually associated with a better thyroid function after the administration of ICM. This initially unexpected and somewhat surprising result can be explained by the fact that patients with subclinical hyperthyroidism were more likely to receive appropriate and timely prophylactic treatment before being administered ICM, and this led to an improvement in their thyroid function afterwards.

As concerns cost-effectiveness, methimazole alone emerged as the best choice for prophylaxis against ICMIH in our selected high-risk sample. This finding should be taken with a degree of caution, however, due to the small number of patients treated with methimazole alone; it would need to be confirmed in larger, randomized studies.

Since the absence of a prophylactic treatment was the only independent predictor of ICMIH, we would like to emphasize the important protective role of prophylaxis in this setting, particularly in fragile and high-risk populations.

We are aware that our study has some significant drawbacks. Its retrospective nature and the small number of patients enrolled mean that further evidence will be needed to confirm our findings on a larger scale. The heterogeneous nature of our sample’s thyroid diseases, comorbidities, and treatments is another important limitation of our study. Also, given the lack of randomization in assigning patients to the different prophylactic treatments, more studies will need to be conducted to obtain solid evidence regarding the use of prophylactic treatment prior to the administration of ICM. On the other hand, a real‐life series of high-risk patients like ours, managed at a single center, may represent a strength of our study, and could also explain the relatively high frequency of ICMIH that we observed. Another strong point concerns our inclusion of patients who already had hyperthyroidism before the ICM-related procedure in order to test the efficacy of the prophylactic treatment even in a real-life clinical and emergency context.

## Conclusion

Administering prophylactic treatment to high-risk patients due to undergo a procedure involving ICM may prevent the onset of ICMIH. This prophylaxis is safe and effective, especially in cardiopathic patients who are more likely to experience severe, and even life-threatening complications of hyperthyroidism. As different types of prophylaxis have proved to be safe and effective, treatment with methimazole alone could be the most cost-effective option.

## Data availability statement

The raw data supporting the conclusions of this article will be made available by the authors, without undue reservation.

## Ethics statement

Ethical review and approval was not required for the study on human participants in accordance with the local legislation and institutional requirements. The patients/participants provided their written informed consent to participate in this study.

## Author contributions

JM and IP contributed to conception and design of the study. JM, IP, SC, CC, MB, BS, IM, and FT organized the database. JM performed the statistical analysis. JM and IP wrote the first draft of the manuscript. JM, IP, and CM wrote sections of the manuscript. All authors contributed to the article and approved the submitted version.
